# Increasing illness severity in very low birth weight infants over a 9-year period

**DOI:** 10.1186/1471-2431-6-2

**Published:** 2006-02-06

**Authors:** David A Paul, Kathleen H Leef, Robert G Locke, Louis Bartoshesky, Judy Walrath, John L Stefano

**Affiliations:** 1 Department of Pediatrics, Section of Neonatology, Christiana Care Health Services, Newark, DE, USA.; 2 Department of Pediatrics, duPont Hospital for Children, Wilmington, DE, USA. Department of Pediatrics, Thomas Jefferson University, Philadelphia, PA, USA; 3 Eugene duPont, Preventive Medicine and Rehabilitation Institute, Christiana Care Health Services, Newark, DE, USA

## Abstract

**Background:**

Recent reports have documented a leveling-off of survival rates in preterm infants through the 1990's. The objective of this study was to determine temporal changes in illness severity in very low birth weight (VLBW) infants in relationship to the outcomes of death and/or severe IVH.

**Methods:**

Cohort study of 1414 VLBW infants cared for in a single level III neonatal intensive care unit in Delaware from 1993–2002. Infants were divided into consecutive 3-year cohorts. Illness severity was measured by two objective methods: the Score for Neonatal Acute Physiology (SNAP), based on data from the 1^st ^day of life, and total thyroxine (T_4_), measured on the 5^th ^day of life. Death before hospital discharge and severe intraventricular hemorrhage (IVH) were investigated in the study sample in relation to illness severity. The fetal death rate was also investigated. Statistical analyses included both univariate and multivariate analysis.

**Results:**

Illness severity, as measured by SNAP and T_4, _increased steadily over the 9-year study period with an associated increase in severe IVH and the combined outcome of death and/or severe IVH. During the final 3 years of the study, the observed increase in illness severity accounted for 86% (95% CI 57–116%) of the variability in the increase in death and/or severe IVH. The fetal death rate dropped from 7.8/1000 (1993–1996) to 5.3/1000 (1999–2002, p = .01) over the course of the study.

**Conclusion:**

These data demonstrate a progressive increase in illness in VLBW infants over time, associated with an increase in death and/or severe IVH. We speculate that the observed decrease in fetal death, and the increase in neonatal illness, mortality and/or severe IVH over time represent a shift of severely compromised patients that now survive the fetal time period and are presented for care in the neonatal unit.

## Background

Despite continuing advances in neonatal care and decades of improving outcomes, it has recently been reported that survival rates have leveled-off in premature infants during the 1990s [1-3]. In the state of Delaware, infant mortality rates improved during the early 1990s similar to national trends, only to see a more recent increase which has been attributed to increasing mortality in very low birth weight infants [4]. In the United States, infant mortality increased in 2002 for the first time in nearly 5 decades [5,6]. In 2002 the United States fetal mortality rate declined while infant mortality increased [6]. A shift from fetal to neonatal deaths is one possible explanation for this trend. The potential impact of increasing fetal survival on neonatal intensive care has not been explored.

Severe intraventricular hemorrhage (IVH) is a significant morbidity in premature infants and is one of the major determinants of long term outcome in very low birth weight infants [7]. Severe IVH has recently shown to have declined in occurrence prior to the 1990's and to have remained unchanged thereafter [8]. Other common morbidities in premature infants including chronic lung disease have also been recently described as increasing in survivors [9]. The reasons for increasing morbidities and recent lack of improvement in survival rates of preterm infants have not been conclusively explained. It has been postulated that the technology for caring for premature infants has reached it limits. One factor that has yet to be investigated is the longitudinal effect of illness severity on morbidity and mortality in premature infants. We hypothesized that illness severity in very low birth weight (VLBW) infants was increasing over time. The objectives of this study were to investigate whether illness was increasing over time and to determine the effect of illness severity on the outcomes of death and/or severe IVH in a population of VLBW infants. As the fetal mortality rate has been declining in the United States [6], and may be leading to a shift from fetal death to physiologically compromised preterm infants, we also explored the fetal death rate at our institution during the study time period.

## Methods

This investigation consisted of a cohort study of VLBW infants, <1500 grams, cared for at a single level III Neonatal Intensive Care Unit (NICU) during a 9-year period, July 1993–July 2002, n = 1414. The NICU at Christiana Hospital is a level III NICU serving the state of Delaware and cares for both inborn (90%) and outborn infants. Neonatal care within the State of Delaware is regionalized. One other level III NICU in the same network serves as a referral center for VLBW infants only if they require surgical care. No other nursery in the State offers intensive care for preterm infants with birth weights <1250 grams. A small number of infants with birthweight between 1250–1500 grams are cared for at two other level II sites within the same state neonatal network. There were no changes in the number of hospitals providing neonatal intensive care during the study time period. The NICU at Christiana Hospital is university affiliated and staffed by residents in pediatrics, neonatal nurse practitioners, and fellows in neonatal/perinatal medicine in addition to attending neonatologists. Decisions concerning resuscitating babies at the limits of viability are made by the attending neonatologist after family consultation. In general, infants 23 to 24 weeks gestation are offered a trial of intensive-care if desired by the family following consultation. This policy did not change during the study period. Throughout the study period Christiana Hospital was the only hospital in the state offering perinatology services and high risk obstetrical care. The Institutional Review Board at Christiana Care Health Services approved this research project. Data were obtained from a computerized database and review of the medical record. Informed written consent was not obtained.

Illness severity was quantified using two objective methods: the Score for Neonatal Physiology (SNAP) [10] and total thyroxine (T_4_). SNAP was routinely calculated after 1996 and therefore was available on infants born after 1996. SNAP was calculated on physiologic data from the 1^st ^24 hours of life. T_4 _was obtained from the State of Delaware Newborn Screening Program and used as a proxy for illness severity. We, and others, have previously shown that total T_4 _is correlated with illness severity in VLBW infants [11-13]. T_4 _was obtained on the 5^th ^day of life as part of routine newborn screening. Therefore, infants who died before the 5^th ^day of life did not have a measurement of T_4_. The mean ± SD of total T_4 _in infants in the State of Delaware during the study period was 13.1 ± 4.2 μg/dl. In order to analyze outcome over time, the study sample was subdivided into consecutive three-year cohorts. *Cohort 1 *included infants born 7/93–7/96, *Cohort 2*, 7/96–7/99, and *Cohort 3*, 7/99–7/02.

Cranial ultrasounds were routinely obtained on the 4^th ^day of life and then monthly until discharge. Cranial ultrasounds were obtained more frequently if clinically indicated. Ultrasounds were done using a 7.5 mHZ transducer and studies were interpreted by a pediatric radiologist. IVH was graded using the classification system of Papile [14]. Severe IVH was considered grade III-IV. Gestational age was based on the best obstetrical estimate. Modified Ballard exam was used only if an obstetrical estimate was not available [15].

A fetal death was defined as any fetus ≥ 20 weeks of gestation that was not live-born. No policy changes in the definition for classifying fetal death were made throughout the study period. Fetal death rate is reported per 1000 births plus fetal deaths. Mothers were classified as receiving steroids if they received any doses of antenatal steroids. Betamethasone is the steroid used routinely at our institution. Clinical chorioamnionitis was diagnosed by the attending obstetrician, based on the presence of fever, uterine tenderness and/or foul smelling amniotic fluid. Any pregnancy with two or more fetuses was considered multiple-gestation. As infertility and in vitro fertilization have been increasing during the study period [16], infertility was investigated as an independent variable. For the purposes of this study, mothers were classified as having a history of infertility if their infants were conceived by in-vitro fertilization or if they received ovulation-enhancing medications such as clomiphene citrate. Mothers were classified as having preeclampsia if given the diagnosis by the attending obstetrician per ACOG guidelines [17]. Oligohydramnios was diagnosed by the attending obstetrician, no criteria for a minimal amniotic fluid index was used as it was not regularly available in the medical record. Prolonged rupture of membranes was considered greater than 24 hours. Maternal age was based on age at time of birth. Infants with birth weight < 1000 grams were analyzed as a sub-group.

Statistical analyses included both univariate and multivariate analysis. Univariate analysis included chi-squared for categorical variables, and analysis of variance for continuous variables with normal distribution. Levene's test of homogeneity of variances was used to assess data distribution. Mann-Whitney U test was used for continuous variables that were not normally distributed. Kruskal-Wallis ANOVA was also used to compare ordinal data over time. Pearson correlation was used to compare two continuous variables. Multivariate analysis included logistic regression and linear regression. The contributions of increased to death and/or severe IVH were calculated by comparing observed to predicted death and or IVH during *Cohort 3*. Data from the logistic regression models were used to calculate a standarized rate [18] for the outcome of death and/or severe IVH. A p value < .05 was considered statistically significant. Statistica (V7.0, Tulsa, OK) was used for all statistical calculations

## Results

A total of 1414 VLBW infants were cared for in the neonatal intensive care unit during the 9-year time period and comprised the study sample. *Cohort 1 *(1993–1996) consisted of 393 infants; there were 485 infants in *Cohort 2 (1996–1999)*, and 536 in *Cohort 3(1999–2002)*. In the study sample, 610 infants (43%) were < 1000 grams birth weight. The proportion of infants with birthweights <1000 grams, 42%, 41%, and 46% (p = .26) in *Cohorts *1, 2, and 3 respectively, did not change over time. The distribution of infants, in 250 gram increments did not differ between Cohorts (Table 1). Of the 1414 infants, 1207 (85%) survived until the 5^th ^day of life and had a measurement of T_4._

**Table 1:** Distribution of birthweights in the study cohorts. Distribution of birthweights did not differ between Cohorts.

	Birthweight <500 grams	Birthweight 500–749 grams	Birthweight 750–999 grams	Birthweight 1000–1249 grams	Birthweight 1250–1500 grams
*Cohort 1 *1993–1996	2%	19%	22%	26%	31%
*Cohort 2 *1996–1999	2%	15%	24%	30%	29%
*Cohort 3 *1999–2002	2%	18%	25%	27%	28%

Birthweight and maternal age were not different between the first, second and third cohorts (Table 2). There was a small but significant decrease in gestational age over time. There was no difference in the percentage of inborn infants over time. There was an increased use of antenatal steroids and antibiotics during the *2*^*nd *^and *3*^*rd *^*Cohorts *compared to the *1*^*st *^*Cohort*. There was also an increased occurrence of preeclampsia and cesarean delivery over time. There were no differences in the occurrence of maternal HELLP syndrome, diabetes, illicit drug use, clinical chorioamnionitis, prolonged rupture of membranes, oligohydramnios, multiple gestation, or infertility over time. In the infants in the study sample, there was no change in the use of mechanical ventilation over time but the use of rescue high frequency ventilation and surfactant did increase over time. Because dopamine can inhibit pituitary hormone production [19], the use of dopamine and dexamethasone in infants was investigated. The use of dopamine in infants increased progressively over time while the use of postnatal dexamethasone increased between *Cohort 1 *and *Cohort 2 *then dropped during *Cohort 3*.

**Table 2:** Changes in demographics and outcomes over time in the overall population.

	*Cohort 1 *1993–1996 n = 393	*Cohort 2 *1996–1999 n = 485	*Cohort 3 *1999–2002 n = 536
Birthweight (g)	1052 ± 297	1057 ± 277	1028 ± 284
Gestational Age (weeks)	28.3 ± 3.0	28.4 ± 2.9	27.8 ± 2.9 * †
Maternal age (years)	26.5 ± 5.9	26.6 ± 6.3	26.7 ± 6.7
Inborn	91%	88%	87%
Apgar score at 5 minutes (median, interquartile range)	8 (6–9)	8 (7–9)	8 (7–9)
Apgar score at 5 minutes <5	13%	8%	10%
Cesarean delivery	55%	57%	62% *
Antenatal steroids	48% ‡	66%	65% *
Antenatal antibiotics	34% ‡	50%	51% *
Preeclampsia	14% ‡	20%	24% *
HELLP syndrome	5%	4%	3%
Oligohydramnios	14%	11%	13%
Clinical chorioamnionitis	9%	7%	8%
Maternal fever	4%	2%	4%
Prolonged rupture of membranes	22%	22%	21%
Illicit maternal drug use	3%	2%	3%
Maternal diabetes	5%	3.5%	5%
Multiple gestation births	24%	24%	28%
Infertility	10%	7%	10%
Mechanical ventilation	78%	79%	83%
Rescue high frequency ventilation	13%	15%	22% *†
Exogenous surfactant replacement	63%	65%	75% *†
Postnatal dopamine	24% ‡	31%	35% *
Postnatal dexamethasone	19% ‡	25%	16% †

*p < .05, Cohort 1v 3

‡p < .05, Cohort 1 vs 2

†p < .05, Cohort 2 vs 3

Postnatal illness severity increased over time, as measured by total T_4 _and SNAP (Table 3). Linear regression models were used to assess the effect of birth cohort on illness severity. Forward stepwise linear regression was used. Variables entered into the model included those that were significant on univariate analysis and variable which are known confounders for illness severity such as birthweight. Birth cohort remained significant in the models when illness severity was measured using SNAP as the dependent variable (model r^2 ^= .41, p < .01) and T_4 _as the dependent variable (model r^2 ^= .35, p < .01). Other than birth cohort, gestational age and birthweight were the only independent variables contributing more to the variability in both models of illness severity. In addition to gestational age and birthweight, the models controlled for maternal antibiotics, multiple gestation birth, race, maternal steroids, preeclampsia, cesarean delivery and inborn status.

**Table 3:** Changes in illness severity and outcomes over time

	*Cohort 1 *1993–1996, n = 393	*Cohort 2 *1996–1999, n = 485	*Cohort 3 *1999–2002, N = 536
T_4 _(μg/dl)	7.6 ± 3.8 ‡	7.0 ± 3.4	6.5 ± 3.9 * †
SNAP	NA	11.2 ± 5.8	13.4 ± 7.6 †
Death	14%	13%	16%
Day of death (median, interquartile range)	7 (2–21)	10.5 (2–22)	3 (2–10) * †
Severe IVH	9%	9%	14% * †
Death and/or Severe IVH	18%	19%	26% * †

*p < .05, Cohort 1vs 3

‡p < .05, Cohort 1 vs 2

†p < .05, Cohort 2 vs 3

There was no difference in the rate of mortality over time (Table 3). However, those infants who died during *Cohort 3*, died earlier compared to those infants who died during both the 1^st ^and 2^nd ^*Cohorts*. There was an observed increase in both severe IVH and the combined outcome of death and/or severe IVH over time. In the sub-group of infants less than 1000 grams, illness severity increased over time as measured by both SNAP and T_4 _(Table 4). Similar to the general study sample, mortality did not increase over time, but there was an increase in severe IVH as well as the outcome of death and/or severe IVH in the infants <1000 g. Table 5 shows changes in T_4 _and SNAP in the study cohorts based on 250 gram birthweight increments. Illness severity decreased with increasing birthweight in each cohort (as measured by increasing SNAP and decreasing T_4_.) Illness severity increased over time in each birthweight subgroup as measured by increasing SNAP. Illness severity, as measured by decreasing T_4_, increased over time in each birthweight subgroup, with the exception of infants 500–750 grams which approached statistical significance.

**Table 4:** Changes in illness severity and outcomes over time in infants <1000 g

Infants <1000 g	*Cohort 1 *1993–1996, n = 165	*Cohort 2 *1996–1999, n = 199	*Cohort 3 *1999–2002, n = 246
T_4 _(μg/dl)	5.4 ± 2.9	5.3 ± 3.1	4.7 ± 3.3 *
SNAP	NA	15.3 ± 7.4	18.3 ± 8.9 †
Death	30%	27%	33%
Severe IVH	16%	14%	23% †
Death/Severe IVH	35%	36%	46% * †

*p < .05, Cohort 1v 3

‡p < .05, Cohort 1 vs 2

†p < .05, Cohort 2 vs 3

**Table 5:** Illness severity by 250 gram birthweight increments. Data for infants <500 grams are not provided due to small numbers.

	Birthweight 500–749 grams	Birthweight 750–999 grams	Birthweight 1000–1249 grams	Birthweight 1250–1500 grams	Birthweight p
**T_4 _(μg/dl)**					
*Cohort 1 *1993–1996	4.5 ± 2.3	6.1 ± 3.1	8.0 ± 3.5	9.8 ± 3.6	<.01
*Cohort 2 *1996–1999	4.3 ± 3.3	5.8 ± 2.9	7.4 ± 2.9	8.9 ± 3.2	<.01
*Cohort 3 *1999–2002	3.6 ± 2.3	5.0 ± 2.9	6.9 ± 3.7	8.3 ± 3.7	<.01
Cohort, p	.20	.03	.04	.03	
**SNAP**					
*Cohort 2 *1996–1999	17.1 ± 6.7	13.6 ± 7.0	10.1 ± 5.0	8.0 ± 4.6	<.01
*Cohort 3 *1999–2002	22.0 ± 9.3	15.8 ± 7.6	11.4 ± 5.4	9.3 ± 5.7	<.01
Cohort, p	.01	.03	.04	.05	

### Effect of Illness on death and/or severe IVH

The combined rate of death and/or severe IVH was 19% in *Cohorts 1 *and *2 *combined. Logistic regression models were created to control for the potential confounding effects of gestational age and birthweight on illness severity. After controlling for gestational age alone by logistic regression, the odds of death and/or severe IVH increased 1.3 (95% CI 1.2–1.5) for every 1 μg/dl drop in total T_4_. When birthweight was added to the model along with gestational age, the odds of death and/or severe IVH increased to a similar degree 1.2 (95% CI 1.1–1.3) for every 1 μg/dl drop in total T_4_. Using T_4 _as a marker for illness severity, the rate of death and/or severe IVH would have been predicted to increase to 25% (95% CI 23–27%) in *Cohort 3*. The observed rate of death and/or severe IVH was 26% (Table 3). If the rate of death and/or IVH would have remained unchanged at 19%, between *Cohorts 1 and 3*, 102 cases of death and/or IVH would have been expected (.19 × 536 infants). In fact, 139 cases of death and/or severe IVH were observed (+37 cases). The observed increase in illness severity would be expected to increase the number of cases of death and/or IVH during *Cohort 3 *by 32 cases (95% CI 19–43 cases). Thus, 32 of the 37, or 86% (95% CI 57–116%) of the observed increase in death and/or severe IVH in *Cohort 3 *could be accounted for by the observed increase in illness severity observed during *Cohort 3*.

Data from the logistic model were also used to calculate a standardized rate [18]. Standardized rate, a tool used for quality assurance, was used to evaluate the possibility that changes in quality of neonatal care were responsible for the observed changes in outcomes. The standardized rate compared the observed rate of death and/or severe IVH to the expected rate of death and or severe IVH based on illness severity, gestational age, and birthweight. Compared to the earlier *Cohorts*, the standardized rate of death and or severe IVH dropped in *Cohort 3 *when illness severity was accounted for using either T_4 _or SNAP [Figure].

### Fetal death rate

During the study period the fetal death rate at Christiana Hospital decreased over time. The fetal death rate was 7.8/1000 in *Cohort 1*, 6.7/1000 in *Cohort 2*, and 5.3/1000 in *Cohort 3 *(p = .01, *Cohort 1 *vs *3*).

## Discussion

Our data show an increase in illness severity in VLBW infants over a 9-year period at a regional level III NICU in the State of Delaware. During this same time period, there was an associated decrease in the fetal death rate, and an increased occurrence in severe IVH. The contribution from increased illness accounted for a majority of the variability of the observed increase in the composite outcome of death and/or severe IVH during the final cohort.

To our knowledge this is the first report of increasing illness severity in a population of VLBW infants. Our report is consistent with data from Vermont-Oxford Network and others who have recently reported no improvement in survival of VLBW infants during the 1990's [1-3]. Although the reasons behind these plateaus in survival rates were not clear, it has been hypothesized that neonatal care has reached its limits. In our population of VLBW infants, the increase in severe IVH was temporally associated with an increase in illness severity as quantified by both SNAP and T_4_. Our data suggest that a majority of the increase in the combined outcome of death and severe IVH could potentially be accounted for by increasing illness. In addition, VLBW infants who died during the *3*^*rd *^*Cohort *(1999–2002), died at an earlier time compared to the first 2 cohorts. The increased illness severity, documented on the 1^st ^day of life by SNAP scores, and the earlier time of death, suggest an antenatal or perinatal etiology for the observed increase in illness.

We speculate that a decrease in fetal death rate has resulted in an increased number of compromised infants and an increase in neonatal illness. Fetal compromise has previously been shown to be associated with neonatal death [20]. In our study sample, there was an increase in cesarean delivery, antibiotics, and steroids over time as has been observed in other recent studies [1,9,21]. Despite these obstetrical interventions, we observed no improvement in survival and increase in severe IVH, one of the major morbidities in VLBW infants over time. Changes in perinatal management of VLBW infants, in an attempt to aggressively care for pregnancies that may have previously been considered non-viable from a medical or gestational age stand-point, may be contributing to the decrease in fetal mortality and subsequent increase in neonatal illness. Alternatively, the observed increase in illness severity may be attributed to other factors, which we were unable to quantify in this study, such as increasing maternal stressors in the antenatal period. The fact that survival rates have remained consistent despite increasing illness suggests that neonatal care may be continuing to advance but not at a rate fast enough to overcome a steady increase in illness severity. Although the VLBW infants in *Cohort 3 *had a similar occurrence of death compared to the older cohorts, increasing illness may be contributing to an increase in neonatal morbidities such as severe IVH in survivors. Consistent with our study, other important neonatal morbidities including bronchopulmonary dysplasia [9], retinopathy of prematurity [22] and neurodevelopmental disabilities [21] have all been recently shown to be increasing in surviving preterm infants.

The present investigation used T_4 _as a proxy for illness as we and others have previously shown the association of T_4 _and illness severity [11-13]. We feel that T_4 _is a valid marker of illness severity as confirmed by the inverse correlation between SNAP and T_4 _in our study sample. Previous research has shown equivalent ROC curves for T_4 _and SNAP in relationship to the outcomes of severe IVH and death [11]. In support of the fact that T_4 _is a marker for illness severity, rather than an important part of the causal pathway, studies of thyroxine supplementation in preterm infants have failed to improve neonatal outcomes [11,23-25]. Because T_4 _was measured on the 5^th ^day of life, we can not rule out the possibility that early management of VLBW infants may have influenced illness severity and T_4_. This possibility, however, remains unlikely as management strategies did not change dramatically at our center during the study period. The use of dopamine, which inhibits thyroid stimulating hormone production [19], did increase over time. However, increasing dopamine use would be expected with an increase in illness and would not explain an increase in SNAP. It also remains a possibility that low T_4 _is an important part of the causal pathway in illness severity and that some primary change in thyroid function over time in our population contributed to the observed increase in illness severity. Even if a primary decrease in T_4 _was responsible for the observed increase in illness, the importance of a progressive decrease in T_4 _is highlighted by the association of hypothyroxinemia with death [26], IVH [27], cystic periventricular leukomalacia [28], and cerebral palsy [29] in VLBW infants.

Our study has a number of other important limitations. First, since our study sample was from a single region our findings may not apply to other populations. Replication of these analyses from other regions would be needed to determine whether the observed increase in neonatal illness is occurring elsewhere. Our study is also limited by the unavailability of SNAP on infants born during the 1^st ^Cohort and the unavailability of T_4 _on those infants who died prior to the 5^th ^day of life. However, those infants who died prior to thyroid screening would have been expected to have an increase in illness severity compared to infant who survived. Thus our data may have potentially underestimated the trend of increasing illness. Because a small number of infants with birth weight between 1250 and 1500 grams are cared for at other centers in the state of Delaware they were not included in this analysis. There were no changes in the number of hospital providing neonatal intensive care or maternal-fetal medicine services during the study duration. Furthermore, the increased illness in our region was present in infants <1000 grams, all of which were captured in this study sample. Because all infants <1000 grams born in the state of Delaware during the study period were captured in our cohorts it is unlikely that any changes in referral patterns influenced the results. Although the number of infants <1500 grams increased in our neonatal intensive care unit over time, the proportion of infants <1000 grams did not change over time. Multivariate modeling also adjusted for changes in gestation over time. We can not conclusively rule out the possibility that head ultrasounds were interpreted differently over time. Our data however are from a single center with uniform ordering of cranial sonograms and definitions of severe IVH. Although we were able to look at many variables influencing illness severity including clinical chorioamnionitis, there may have been other variables such as histologic chorioamnionitis [30] or time of birth [31] which we were unable to explore. Finally, although also unlikely, we can not rule out a change in the quality of obstetrical or neonatal care as a cause for increasing death on/or severe IVH. The similarities in the distribution of Apgar scores over time do not suggest that any changes in early delivery room management are responsible for the observed outcomes. The drop in the standardized rate observed over time suggests an improvement in neonatal outcomes, given the magnitude of increased illness severity.

Recent national data have shown that mortality in VLBW infants is no longer declining despite advances in neonatal care [1-3]. Our data are important in showing an increase in illness severity over a 9-year period in VLBW infants. We were also able to document an associated decrease in the fetal death rate during this same period. Infant mortality in the United States has been reported to have increased for the 1^st ^time since 1958 [5]. Similar to our regional findings, the recent increase in infant mortality in the United States was associated with a decrease in the fetal death rate [5]. Based on our data we can not determine the cause of the observed increase in illness in VLBW infants, but speculate that there maybe a shift of fetal deaths to live, but severely physiologically compromised newborns. Future investigations and other regional data analysis will be necessary to confirm these findings.

## List of abbreviations

IVH Intraventricular hemorrhage

NICU Neonatal Intensive Care Unit

SNAP Score for neonatal acute physiology

T_4 _Total thyroxine

VLBW Very low birth weight

## Competing interests

The author(s) declare that they have no competing interests.

## Authors' contributions

DP was responsible for conceiving the design of the study, acquiring the data and maintaining the database, data analysis, drafting and revising the manuscript. KL was responsible for acquiring data, maintaining the database, and manuscript revision. RL participated in data analysis, design of the study, and manuscript revision. LB was responsible for acquiring the data on newborn thyroid function, interpreting data and manuscript revision. JW participated in data analysis and manuscript revision. JS participated in data analysis, design of the study, and manuscript revision. All authors read and approved the final manuscript.

**Figure 1:**
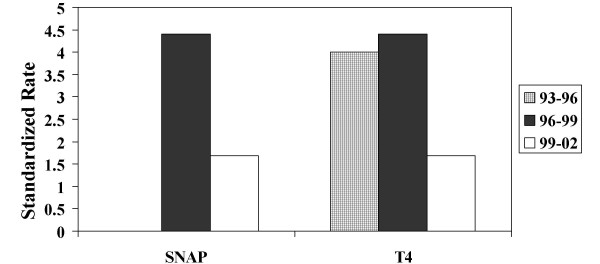
Standardized rate for death and/or severe IVH. Standardized rate is a ratio of observed/expected rate of death and/or severe IVH. Expected rate of IVH was calculated using logistic regression models (see text) using either SNAP or T_4 _to adjust for illness severity. Checkered box indicates 1993–1996, black boxes 1996–1999, white boxes 1999–2002.

## Pre-publication history

The pre-publication history for this paper can be accessed here:



## References

[B1] Horbar JD, Badger GJ, Carpenter JH, Fanaroff AA, Kilpatrick S, LaCorte M, Phibbs R, Soll RF (2002). Trends in mortality and morbidity for very low birth weight infants, 1991-1999. Pediatrics.

[B2] Kaiser JR, Tilford JM, Simpson PM, Salhab WA, Rosenfeld CR (2004). Hospital survival of very-low-birth-weight neonates from 1977 to 2000. J Perinatol.

[B3] Meadow W, Lee G, Lin K, Lantos J (2004). Changes in mortality for extremely low birth weight infants in the 1990s: implications for treatment decisions and resource use. Pediatrics.

[B4] (2003). Increasing infant mortality among very low birthweight infants--Delaware, 1994-2000. MMWR Morb Mortal Wkly Rep.

[B5] Kochanek KD, Smith BL (2004). Deaths: preliminary data for 2002. Natl Vital Stat Rep.

[B6] Martin JA, Kochanek KD, Strobino DM, Guyer B, MacDorman MF (2005). Annual summary of vital statistics--2003. Pediatrics.

[B7] Vollmer B, Roth S, Baudin J, Stewart AL, Neville BG, Wyatt JS (2003). Predictors of long-term outcome in very preterm infants: gestational age versus neonatal cranial ultrasound. Pediatrics.

[B8] Fanaroff AA, Hack M, Walsh MC (2003). The NICHD neonatal research network: changes in practice and outcomes during the first 15 years. Semin Perinatol.

[B9] Hintz SR, Poole WK, Wright LL, Fanaroff AA, Kendrick DE, Laptook AR, Goldberg R, Duara S, Stoll BJ, Oh W (2005). Changes in mortality and morbidities among infants born at less than 25 weeks during the post-surfactant era. Arch Dis Child Fetal Neonatal Ed.

[B10] Richardson DK, Gray JE, McCormick MC, Workman K, Goldmann DA (1993). Score for Neonatal Acute Physiology: a physiologic severity index for neonatal intensive care. Pediatrics.

[B11] Paul DA, Leef KH, Voss B, Stefano JL, Bartoshesky L (2001). Thyroxine and illness severity in very low-birth-weight infants. Thyroid.

[B12] van Wassenaer AG, Kok JH, Dekker FW, de Vijlder JJ (1997). Thyroid function in very preterm infants: influences of gestational age and disease. Pediatr Res.

[B13] Huang CB, Chen FS, Chung MY (2002). Transient hypothyroxinemia of prematurity is associated with abnormal cranial ultrasound and illness severity. Am J Perinatol.

[B14] Papile LA, Burstein J, Burstein R, Koffler H (1978). Incidence and evolution of subependymal and intraventricular hemorrhage: a study of infants with birth weights less than 1,500 gm. J Pediatr.

[B15] Ballard JL, Khoury JC, Wedig K, Wang L, Eilers-Walsman BL, Lipp R (1991). New Ballard Score, expanded to include extremely premature infants. J Pediatr.

[B16] Wright VC, Schieve LA, Reynolds MA, Jeng G, Kissin D (2004). Assisted reproductive technology surveillance--United States, 2001. MMWR Surveill Summ.

[B17] (2002). ACOG practice bulletin. Diagnosis and management of preeclampsia and eclampsia. Number 33, January 2002. American College of Obstetricians and Gynecologists. Int J Gynaecol Obstet.

[B18] Richardson D, Tarnow-Mordi WO, Lee SK (1999). Risk adjustment for quality improvement. Pediatrics.

[B19] Van den Berghe G, de Zegher F, Lauwers P (1994). Dopamine suppresses pituitary function in infants and children. Crit Care Med.

[B20] Batton DG, DeWitte DB, Espinosa R, Swails TL (1998). The impact of fetal compromise on outcome at the border of viability. Am J Obstet Gynecol.

[B21] Wilson-Costello D, Friedman H, Minich N, Fanaroff AA, Hack M (2005). Improved survival rates with increased neurodevelopmental disability for extremely low birth weight infants in the 1990s. Pediatrics.

[B22] Hameed B, Shyamanur K, Kotecha S, Manktelow BN, Woodruff G, Draper ES, Field D (2004). Trends in the incidence of severe retinopathy of prematurity in a geographically defined population over a 10-year period. Pediatrics.

[B23] Biswas S, Buffery J, Enoch H, Bland M, Markiewicz M, Walters D (2003). Pulmonary Effects of Triiodothyronine (T(3)) and Hydrocortisone (HC) Supplementation in Preterm Infants less than 30 Weeks Gestation: Results of the THORN Trial-Thyroid Hormone Replacement in Neonates. Pediatr Res.

[B24] Smith LM, Leake RD, Berman N, Villanueva S, Brasel JA (2000). Postnatal thyroxine supplementation in infants less than 32 weeks' gestation: effects on pulmonary morbidity. J Perinatol.

[B25] van Wassenaer AG, Kok JH, de Vijlder JJ, Briet JM, Smit BJ, Tamminga P, van Baar A, Dekker FW, Vulsma T (1997). Effects of thyroxine supplementation on neurologic development in infants born at less than 30 weeks' gestation. N Engl J Med.

[B26] Paul DA, Leef KH, Stefano JL, Bartoshesky L (1998). Low serum thyroxine on initial newborn screening is associated with intraventricular hemorrhage and death in very low birth weight infants [see comments]. Pediatrics.

[B27] Paul DA, Leef KH, Stefano JL, Bartoshesky L (2000). Thyroid function in very-low-birth-weight infants with intraventricular hemorrhage [In Process Citation]. Clin Pediatr (Phila).

[B28] Leviton A, Paneth N, Reuss ML, Susser M, Allred EN, Dammann O, Kuban K, Van Marter LJ, Pagano M (1999). Hypothyroxinemia of prematurity and the risk of cerebral white matter damage. J Pediatr.

[B29] Reuss ML, Paneth N, Pinto-Martin JA, Lorenz JM, Susser M (1996). The relation of transient hypothyroxinemia in preterm infants to neurologic development at two years of age [see comments]. N Engl J Med.

[B30] De Felice C, Toti P, Parrini S, Del Vecchio A, Bagnoli F, Latini G, Kopotic RJ (2005). Histologic chorioamnionitis and severity of illness in very low birth weight newborns. Pediatric Critical Care Medicine.

[B31] Lee SK, Lee DS, Andrews WL, Baboolal R, Pendray M, Stewart S, Canadian Neonatal N (2003). Higher mortality rates among inborn infants admitted to neonatal intensive care units at night.[see comment]. Journal of Pediatrics.

